# Cracking the code to female sexual satisfaction: the serial mediation of sexual behavior and the perceived importance of healthy sexuality from sexual self-efficacy

**DOI:** 10.3389/fpsyg.2024.1305399

**Published:** 2024-05-17

**Authors:** Adelaida Irene Ogallar-Blanco, Raquel Lara-Moreno, Raquel García-Pérez, Antonio Liñán-González, Débora Godoy-Izquierdo

**Affiliations:** ^1^Departamento de Personalidad, Evaluación y Tratamiento Psicológico, Facultad de Psicología, University of Granada, Granada, Spain; ^2^Departamento de Psicología Social, Facultad de Psicología, University of Granada, Granada, Spain; ^3^Departamento de Enfermería, Facultad de Ciencias de la Salud, Parque Tecnológico de la Salud, University of Granada, Granada, Spain; ^4^Departamento de Enfermería, Facultad de Ciencias de la Salud, University of Granada, Melilla, Spain

**Keywords:** sexual satisfaction, sexual self-efficacy, sexual behavior, sexual health, females’ sexuality

## Abstract

**Introduction:**

Sexual satisfaction has been shown to have a strong association with many aspects of sexual health and wellbeing. It is further considered a robust indicator of an individual’s health status and general wellbeing, revealing that a person can enjoy pleasurable and healthy sexual experiences, beyond the mere absence of sexual and reproductive health issues.

**Objectives:**

This study aimed to analyze the relationship between sexual satisfaction, sexual behaviors, sexual self-efficacy, and the importance personally attributed to maintaining an active and satisfying sexual life among young and middle-aged women aged 18–50.

**Design:**

A descriptive correlational study with a cross-sectional design was conducted.

**Methods:**

Participants (*N* = 1,076 women) completed self-reports on sexual self-efficacy beliefs, frequency of sexual behaviors, the importance attributed to active and healthy sexuality, and multidimensional sexual satisfaction.

**Results:**

The supported mediation model indicated that sexual self-efficacy was related to sexual satisfaction directly and indirectly through sexual behavior and a serial path through sexual behavior and the perceived importance of healthy sexuality. The total effect was significant, and the full model explained 7.3% of the global sexual satisfaction variance (*F* = 17.218, *p* = 0.000), with the mediated effect accounting for 44.3%.

**Conclusion:**

This study confirms a partial serial mediation model by which sexual self-efficacy significantly predicts sexual satisfaction through sexual behaviors and the importance attributed to a healthy sexuality. Due to its significant contribution, the perceived importance of sexuality should be considered when studying correlates of sexual satisfaction. These findings have interesting implications for the development of strategies aimed at sexual health promotion and sexual education among women in early and middle adulthood.

## 1 Introduction

A substantial body of evidence supports the relationship between having a satisfying sexual life and various facets of psychosocial wellbeing, including the capacity for love, adjustment and relationship satisfaction, self-esteem, physical and mental health, quality of life, happiness, and overall life satisfaction (e.g., Sánchez-Fuentes et al., 2014; Schmiedeberg et al., 2017; Holdsworth et al., 2018; Van den Brink et al., 2018; Carcedo et al., 2020). Given the positive consequences of sexual satisfaction, it is considered a robust indicator of health status and general wellbeing (Carrobles et al., 2011).

Regarding sexual health, evidence suggests that sexual self-efficacy (SSE) is associated with the adoption of protective methods (e.g., Mahat et al., 2014), especially condom use (e.g., Volkmann et al., 2014; Escribano et al., 2015). Moreover, SSE has been linked to partner communication (Widman et al., 2014); dating violence prevention (e.g., Baker et al., 2014); and the propensity to engage in both high-risk behaviors and health promotion behaviors (e.g., Jones et al., 2016). Most of the research exploring the relationship between self-efficacy beliefs and sexual health has focused on investigating SSE for engaging in preventive behaviors (e.g., declining unprotected sex or negotiating condom use). To the best of our knowledge, relatively little attention has been directed toward investigating SSE for health-promotion behaviors, particularly those aimed at enhancing sexual satisfaction (e.g., including sexual fantasies or erotism in the relationship, practicing new techniques, expressing desires, or initiating a sexual relationship).

For instance, SSE has been found to mediate the relationship between partner trust and condom use (Goodman et al., 2016). Additionally, it plays a mediating role in the link between enacted social support and women’s perceived quality of relationship alternatives (e.g., developing a safety plan) (Tirone et al., 2014), and in the inverse relationship between body objectification, inauthenticity, and condom use (Impett et al., 2006). Recently, SSE has also been found to mediate the relationship between sexual education (i.e., higher-quality sexual education has been shown to enhance SSE) and sexual satisfaction (Nurgitz et al., 2021), while its effect on sexual behaviors has been found to be moderated by other variables such as resilience (Logie et al., 2018).

Concerning the relationship between sexual behavior and its outcomes, considerable evidence supports the role for sexual satisfaction (e.g., Sánchez et al., 2012; Ogallar Blanco et al., 2017; Hawkins et al., 2022). Sexual satisfaction is not only linked to the frequency and diversity of sexual activities or the consistency of achieving orgasm but also to behaviors associated with intimacy, mood, and communication within intimate or sexual relationships (Fisher et al., 2015; Frederick et al., 2017; Schmiedeberg et al., 2017, Schoenfeld et al., 2017). Although the existing literature lacks studies exploring the mediating effects of sexual behavior in the link between SSE and behavioral outcomes, such as sexual satisfaction, we consider this mediation role to be plausible, given the recognized role of self-efficacy perceptions as key predictors of intention and action in health-related behaviors (e.g., Sheeran et al., 2016).

Additionally, the significance attributed to cultivating an active and satisfying sexual life—or sexuality importance—could potentially increase the likelihood of enjoying satisfactory sexual relations and might correlate with the individuals’ intention to adopt healthy behaviors. The importance of sexuality and sexual activity for an individual’s wellbeing is becoming more widely recognized (e.g., Stark et al., 2015; Kolodziejczak et al., 2021) and is being investigated as a key factor in women’s sexuality and quality of life across various life stages (e.g., Thomas et al., 2015; Juang and Knight, 2018; Kolodziejczak et al., 2021) and health conditions (e.g., Salakari et al., 2020).

Despite the growing recognition of research on SSE, sexual satisfaction, sexual behavior, and the importance of maintaining an active and satisfying sexual life, studies examining the interaction between all these variables remain scarce (e.g., Sánchez et al., 2012; Rausch and Rettenberger, 2021). Furthermore, a notable limitation in the existing literature is the narrow age focus, with many studies concentrating on specific age groups such as adolescents or menopausal women (e.g., Rausch and Rettenberger, 2021; Vasconcelos et al., 2021), with broader age spans less frequently investigated. The present study addresses this gap by studying these variables in women aged 18–50, an important age range encompassing crucial life periods. This age range is marked by significant milestones, such as navigating, exploring, and establishing personal desires, preferences, and attitudes toward sexuality (e.g., sexual orientation, identity, and self-knowledge), the growth of intimate relationships, and the potential impact of factors such as pregnancy, childbirth, and menopause on sexual health, all of which can influence sexual activity and satisfaction. This is especially important in the Spanish context because these cohorts have experienced the sociocultural changes stemming from the implementation of democracy in this nation. By comprehensively studying the interplay between these factors, we can enhance our understanding of women’s diverse experiences of sexuality.

**FIGURE 1:**
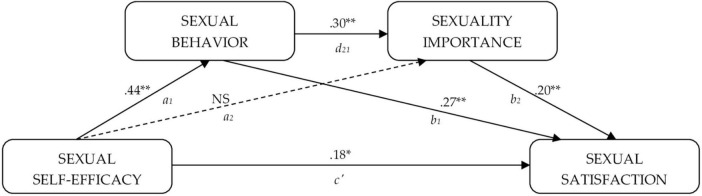
Serial mediation effect of sexual behavior and sexuality importance between sexual self-efficacy and sexual satisfaction (two-mediator serial multiple model type 6). Standardized coefficients. **p < 0.01, *p < 0.05.

In this context, this study explores the relationship between sexual behavior and sexual satisfaction, delving into the influential role of the perceived importance of maintaining an active and satisfactory sexual life and particularly the impact of SSE beliefs on such relationships among women in early and middle adulthood. Our aim was to analyze whether the relationship between sexual self-efficacy and sexual satisfaction could be mediated by sexual behavior and the importance attributed to maintaining an active and satisfactory sexual life. Figure 1 displays the relationship tested. We expected to find a positive relationship between the variables as well as an indirect, mediated effect indicating the role of sexual behavior and attitudes toward sexuality in translating self-efficacy beliefs into actions, ultimately influencing satisfaction with sexuality.

## 2 Materials and methods

### 2.1 Participants

An initial sample (*N* = 1,877) of volunteers was recruited through non-probabilistic sampling. Eleven cases were eliminated due to methodological criteria (e.g., test cases), 512 because they had not responded to the main measures, and 274 were excluded for not meeting the inclusion criteria. The latter included self-identified female gender, aged between 18 and 50 years, being resident in Spain for at least 1 year, proficiency in reading and writing in Spanish, and the absence of severe mental/physical disease. The age range was selected based on the rationale that these cohorts have witnessed the sociocultural developments resulting from the establishment of democracy in Spain, and they were not peri- or post-menopausal women (Dam et al., 2019; Larroy et al., 2020), since these conditions are recognized as key factors influencing female sexuality. The only exclusion criterion applied was the presence of chronic or severe medical conditions that could significantly impact sexual functioning. Additionally, four outlier cases were identified and removed during preliminary analyses.

The final sample consisted of 1,076 women aged 18–50 years (*M* = 24.47; SD = 6.11). Of these, 1,046 (97.2%) were Spanish citizens born and raised in Spain, and 30 were European (1.8%), Latin-American (0.8%), or Asian (0.2%). Of these, 94.3% had spent their entire lives in Spain, 1.8% more than 20 years, 2.5% between 11 and 20 years, 0.6% between 6 and 10 years, and 0.8% between 1 and 5 years. As a result, the included foreigners reported having spent most of their lives in Spain (98.6%), minimizing potential cultural influences. Additionally, 80.4% were heterosexual, 3.2% lesbian, 13.1% bisexual, and 3.3% had not yet defined their sexual orientation. Moreover, 68.9% had a sexual partner(s). Other participant characteristics refer to their educational level, religiosity, and experiences within sexual/intimate relationships (Table 1).

**TABLE 1:** Socio-demographic and personal data (*N* = 1,076).

	*N*	%
**Educational level**		
Primary school	13	1.2
Secondary school	23	2.1
Professional training	53	4.9
University	859	79.8
University postgraduate (master/PhD)	128	11.9
**Religion**		
Catholic	475	44.1
Other (Buddhist, Muslim, Protestant, not indicated)	20	2
Agnostic	149	13.8
Atheist	432	40.1
**Religiosity grade**		
Non-believer (not religious at all)	628	58.4
Something believer (rather not religious)	287	26.7
Quite believer (rather religious)	119	11.1
Very believer (very religious)	42	3.8
**Sexual/romantic partner at the time of the study**		
In a relationship for less than 1 year	177	16.5
In a relationship between 1 and 5 years	321	29.9
In a relationship for more than 5 years	242	22.5
Not in a relationship or with no sexual partner(s)	336	31.1

### 2.2 Measures

(A) Sexual self-efficacy: The original Spanish version of the Sexual Self-Efficacy Questionnaire (Ogallar-Blanco et al., 2023) was used. This bi-dimensional measure includes 10 items measuring SSE for preventive actions [e.g., “*How confident in yourself are you when discussing sexually transmitted disease prevention (e.g., AIDS, herpes) with your sexual partner(s)?*”] and 10 items for health promotion actions [e.g., “*How confident in yourself are you when discussing what you like or would like to do during sex (new techniques, different situations or places, use of toys, etc.) with your partner(s)?*”], all answered on a 5-point Likert-type scale (0 = not at all confident; 4 = very confident). Average values were calculated, with higher values indicating higher SSE. The *alpha* value was 0.90. Appropriate psychometric properties have been reported for this measure, which obtained and *alpha* value of 0.89 for the complete questionnaire (Ogallar-Blanco et al., 2023).
(B) Sexual behaviors: These were assessed using a self-report questionnaire concerning the frequency of 25 partner activities (e.g., kissing and intercourse) and individual behaviors (e.g., masturbation and fantasy). Responses were provided on a 4-point Likert-type scale (0 = never; 3 = very frequently). Additional items related to sexuality included the frequency of engaging in fantasies (0 = never; 3 = always), taking the initiative within the relationship (0 = always my partner; 4 = always me), communication (0 = no, never; 4 = always), and use of contraception methods (0 = never; 5 = always) (e.g., “*When you and your partner have sex, who usually takes the initiative, who makes the first step, or suggests doing something sexual?*”). Given the asymmetry in the response scale, a summation variable was computed, where a higher value indicated a greater frequency of the assessed sexual behaviors. All items were derived from expert judgments and professional and research sources (e.g., Levay and Valente, 2006), with similar frequency tools having been employed in previous studies for these purposes (e.g., Carrobles et al., 2011; Katz and Schneider, 2015).
(C) Sexuality importance: To assess the perceived importance of having an active and satisfying sexual life, participants responded to a face-valid question (“*How important is it for you to have an active, healthy, and satisfying sexuality?*”) on a 4-point Likert-type scale (0 = not important at all; 3 = very important). Higher values indicated a greater perceived importance. Similar one-single indicators have been used in previous studies (Thomas et al., 2015; Kolodziejczak et al., 2021).
(D) Sexual satisfaction was assessed using the original Spanish version of the Sexual Satisfaction Comprehensive Index, Ogallar-Blanco et al. (2022) including four face-valid items regarding the actual and desired sexual satisfaction while performing partnered and individual sexual activities [e.g., “*How satisfactory are your sexual relationships with a partner(s), in general? (If you don’t have any sexual partner(s) currently think about your past relationships in general)*”], rated on a 4-point Likert-type scale (0 = Not satisfactory at all/Not interested; 3 = Very satisfactory). An average score was calculated to obtain a global sexual satisfaction indicator. This score reflects the participant’s satisfaction with her current sexual experiences and the extent to which this diverges from expected levels of satisfaction, both with or without a sexual partner. A higher value on this indicator signifies greater levels of sexual satisfaction. The *alpha* value was 0.94. Appropriate psychometric properties have been reported for this scale, obtaining a total *alpha* value of 0.89 (Ogallar-Blanco et al., 2022).

Other socio-demographic characteristics and sexual experiences of the participants are shown in Table 1. The assessed factors included sex-gender, age, sexual orientation, educational level, religion, degree of religiosity, current romantic partner status, presence of any severe disease at the time of the study, nationality, place of residence, duration of residence in Spain, and proficiency in the Spanish language.

### 2.3 Procedure

Participants were recruited using non-probabilistic, targeted sampling. The online assessment protocol (LimeSurvey^®^, LimeSurvey GmbH, Germany) was publicized online (e.g., social networks of professionals of Psychology and Sexology, social media) and through traditional media (e.g., direct requests for participants to share or participate, snowball procedure) to recruit a nationwide sample. This survey was accessible from November 2021 to February 2023. Participants included women who voluntarily accessed and completed the online survey. They were informed about their rights, the characteristics of the study, and the confidentiality of their responses. Only after informed consent was given could they access the survey. Neither feedback nor compensation was offered. Ethical approval was obtained from the Ethics Committee on Human Research of the University of Granada, reg. CEFM-44521-0511.

### 2.4 Study design and data analyses

This is a descriptive, correlational study with a cross-sectional design.

Preliminary and exploratory data analyses were conducted to detect (and correct) possible errors in data entry, missing data, or outliers. Four multivariate outliers (Mahalanobis’ distance test) were detected and removed (*N* = 1,076). No univariate outliers (boxplot) were identified. After checking assumptions of normality and homoscedasticity, we conducted descriptive and Pearson’s correlation analyses.

Additionally, analyses of indirect mediation effects were conducted to establish the degree to which a predictor variable influences an outcome through one or more mediator variables in a causal model (Hayes, 2009). Mediation is confirmed when the predictor affects the outcome indirectly through at least one intervening or *process* variable (Baron and Kenny, 1986; Preacher and Hayes, 2004, 2008; MacKinnon et al., 2007). To examine possible indirect effects, two-mediator serial multiple mediation analyses were conducted using the “PROCESS” macro for SPSS v.28 (Hayes, 2012, 2013) using a non-parametric resampling method with bootstrapping (MacKinnon et al., 2004; Preacher and Hayes, 2004, 2008; Hayes, 2009). Resampling of the data was carried out by creating 5,000 random samples for parameter estimation, ensuring the stability of the analysis. Corrected 95% confidence intervals were calculated for the distribution of the *ab* and *adb* coefficients obtained by resampling.

Mediation analyses were conducted with both raw and standardized scores to convert the variables to a comparable measurement scale (Marquardt, 1980). Standardization of the data is recommended in mediation analyses (Cheung, 2009) because it allows researchers to generate standardized indirect effects within the range 0 to ±1. This standardization makes the effects readily interpretable and comparable, serving as an estimate of effect sizes and allowing comparisons between studies. The effect size, or standardized indirect effect, provides a measure (independent of the variable measurement scales) of the direction and magnitude or strength of the association between the variables. In the case of mediation analysis, it enables the formulation of the equation: (standardized) total effect = (standardized) direct effect + (standardized) indirect effects (c = c′ + ab_1_ + ab_2_ + a_1_d_21_b_2_). In contrast, because effect size depends directly on the scale of the variables, the (non-standardized) effects estimated from the raw data allow only the calculation of their statistical significance.

Analyses were conducted with SPSS 28.0 and AMOS 22 (SPSS BMI Inc., Chicago, IL, USA). The level of significance was set at *p* < 0.05 for all analyses.

## 3 Results

Table 2 provides an overview of the descriptive results. The mean value for SSE was notably high, and the standard deviation indicated a high degree of homogeneity among the participants. The mean score for sexual behaviors was moderate, with a corresponding between-subject variability. Both sexuality importance and sexual satisfaction yielded high mean scores, with substantial homogeneity between the participants, particularly in the case of sexual satisfaction. Pearson’s order-zero correlation analyses (Table 2) indicated that all the variables were positively intercorrelated.

**TABLE 2:** Descriptive data and bivariate zero-order correlations for the study variables.

Variable (scores range)	*M* ± SD [min–max]	2	3	4
1. Sexual self-efficacy average (0–4)	3.37 ± 0.53 [0–4]	0.36**	0.16**	0.30**
2. Sexual behavior summation (0–91)	43.56 ± 12.46 [14–88]	–	0.35**	0.40**
3. Sexuality importance (0–3)	2.19 ± 0.71 [0–3]		–	0.25**
4. Global sexual satisfaction (0–3)	2.47 ± 0.44 [0.5–3]			–

**p < 0.01.

A two-mediator multiple serial analysis (Model 6 of PROCESS with 2 mediators operating serially) was conducted to explore whether sexual behavior and/or sexuality importance could be paths of interest (mediator variables) in the relationship between SSE (predictor variable) and sexual satisfaction (outcome variable) (Figure 1). SSE predicted sexual satisfaction directly and indirectly through the mediator variable sexual behaviors alone (indirect effect 1: ab_1_) and the path sexual behavior-sexuality importance (indirect effect 2: a_1_d_21_b_2_). Consequently, the stronger the SSE, the higher the frequency/type of sexual behaviors, and the greater the level of overall sexual satisfaction (indirect effect 1). Additionally, the stronger the SSE, the higher the frequency/type of sexual behaviors, and the greater the perceived importance of sexuality, resulting in greater overall sexual satisfaction (indirect effect 2). The path for sexuality importance alone (indirect effect 3: ab_2_) was not significant. The total effect was significant, and the full model explained 7.3% of the overall sexual satisfaction variance, *F* = 17.218, *p* = 0.000. The mediated effect accounts for 44.3% of the total effect. Thus, a simple and a serial partial mediation effect was confirmed in which SSE predicts sexual satisfaction, both directly and indirectly, through sexual behaviors alone and through sexual behaviors and the perceived importance of maintaining an active and satisfactory sexual life (see Table 3). The same analyses were conducted using age (beta = −0.00, *p* = 0.890) and religiosity level (beta = −0.20, *p* = 0.070) as covariates of the mediators or of the outcome sexual satisfaction, but neither showed significance or changed the findings reported above (*F* = 7.593; *p* = 0.000).

**TABLE 3:** Results of two-mediators’ multiple serial analyses for *Z* scores (with Process).

Effect	Standardized coefficient	SE	*t*	*p*	95% CI LL	95% CI UL
a_1_ (SSE – SB)	0.4380	0.0765	5.7250	0.0000	0.2872	0.5888
a_2_ (SSE – SI)	−0.0133	0.0704	−0.1883	0.8508	−0.1520	0.1255
b_1_ (SB – SS, controlling for SSE)	0.2653	0.0661	4.0111	0.0001	0.1349	0.3957
b_2_ (SI – SS, controlling for SSE)	0.1988	0.0731	2.7192	0.0071	0.0547	0.3430
d_21_ (SB – SI, controlling for SSE)	0.2927	0.0581	5.0371	0.0000	0.1781	0.4072
c′ (direct effect SSE – SS, controlling for SB and SI)	0.1752	0.0758	2.3099	0.0218	0.0257	0.3246
ab_1_ (indirect effect SSE × SB, c-c′)	0.1162	0.0389			0.0540	0.2066
ab_2_ (indirect effect, SSE × SI, c-c′)	−0.0026	0.0157			−0.0392	0.0248
a_1_d_21_b_2_ (indirect effect SSE × SB × SI, c-c′)	0.0255	0.0125			0.0079	0.0600
c (total effect SSE – SS, c′ + ab_1_ + ab_2_ + a_1_d_21_b_2_)	0.3142	0.0757	4.150	0.0000	0.1650	0.4635

PROCESS Model 6. Bootstrapping: 5,000 samples. SE, standard error; 95% CI, corrected 95% confidence intervals; LL, lower limit; UL, upper limit; SSE, sexual self-efficacy; SB, sexual behaviors; SI, importance of sex and sexuality; SS, sexual satisfaction.

## 4 Discussion

This study analyzed the relationship between SSE beliefs regarding healthy sexuality, sexual behaviors, perceived importance of an active and satisfying sexuality, and sexual satisfaction among a cohort of young and middle-aged adult women. Sexual behaviors and sexuality importance were serial mediators in the link between SSE and sexual satisfaction, which were also directly related.

The study participants were a homogenous group with a satisfactory sexual life and high SSE. However, the diversity of sexual behaviors was less varied among participants. Furthermore, the perceived importance of maintaining an active, healthy, and satisfying sexual life was consistently high. Our findings revealed that higher levels of sexuality importance and increased variety and frequency of sexual behaviors were associated with greater SSE, and in turn with higher levels of satisfaction. This association seems reasonable, given that a broader range of sexual behaviors could foster an increased interest in achieving greater sexual satisfaction, consequently increasing the importance assigned to maintaining an active and satisfactory sexuality (Thomas et al., 2015; Schmiedeberg et al., 2017; Rausch and Rettenberger, 2021). Furthermore, our findings suggest that if a woman places a higher value on her sexuality, she is likely to engage in more frequent and varied behaviors driven by a proactive approach to avoid potential undesired consequences (e.g., unwanted pregnancies and sexual dysfunctions) and to enhance sexual pleasure (e.g., seeking information and communicating with a partner). Moreover, the association between sexuality importance and the frequent and flexible practice of sexual behaviors could contribute to reinforcing SSE beliefs. For instance, a woman’s interest in having a healthy sexual life could prompt her to seek information about contraceptive methods, forming the basis of preventive behaviors and concurrently strengthening SSE beliefs regarding the successful use of protection methods. All these factors contribute to a more satisfying sexuality. Future research should analyze the relationships between sexuality importance, sexual behaviors, and SSE, with a more in-depth exploration of the participants’ knowledge, attitudes, self-esteem, and motivations.

As expected, our study revealed a positive association between SSE and sexual behaviors, aligning with the principles of Self-efficacy Theory (Bandura, 1982, 1997). According to this theory, stronger SSE beliefs are conducive to engaging in healthier and more active sexual behaviors, and such pleasurable and healthy sexual experiences also reinforce SSE through an enactive mastery experience mechanism (Bandura, 1997). This finding coincides with other investigations (Sheeran et al., 2016). Regarding the participants’ sexual behaviors, while the frequency and variety were not exceptionally high, they appear to be sufficient to ensure more than acceptable levels of sexual satisfaction. The contribution of sexual activities to sexual satisfaction has also been supported elsewhere (McNulty et al., 2016; Schoenfeld et al., 2017; Velten and Margraf, 2017), and our findings are consistent with these previous results. As a result, it is conceivable that individuals, despite not possessing a complex behavioral repertoire, may engage in more successful and satisfying behaviors in various sexual contexts due to their high perceived skills in successfully navigating their sexuality and strong SSE for having an active and healthy sexuality. This in turn may contribute to the reinforcement of their SSE and overall sexual satisfaction.

Given the relationships found between SSE, sexual behaviors, perceived importance assigned to sexuality, and sexual satisfaction, we proceeded to analyze the mechanisms that could explain such associations through an indirect effect (*how* it operates or mediation effects) (Hayes, 2012). Thus, a two-mediator multiple serial mediation model was established to predict overall sexual satisfaction (i.e., individual and partnered actual and desired sexual satisfaction) while considering sexual behaviors and sexuality importance in relation to SSE. The results revealed that SSE was associated with sexual satisfaction both directly and indirectly, through sexual behaviors and through the path of sexual behaviors-sexuality importance. This second indirect effect was found to be the strongest.

No previous investigation has analyzed this *process* relationship between the variables of the present study. Nevertheless, given the ample support for the relationships between SSE and sexual behaviors (e.g., Closson et al., 2018; Logie et al., 2018, Torregosa and Patricio, 2022), between behavior and its consequences, such as sexual satisfaction (e.g., Sachs-Ericsson et al., 2011; Rausch and Rettenberger, 2021), and between SSE and sexual satisfaction (e.g., Assarzadeh et al., 2021; Nurgitz et al., 2021), our results appear to be reasonably consistent with previous findings.

Several studies have explored the impact of variables comparable to the perceived importance of healthy sexuality (e.g., Haavio-Mannila and Kontula, 1997; Thomas et al., 2015; Juang and Knight, 2018; Kolodziejczak et al., 2021). Although these studies did not specifically address the perceived importance of sexual health and satisfaction but referred more generally to the importance assigned to sex or sexuality, we believe that the similarity of the terms makes the results congruent. In essence, both the perceived importance of sex as a behavior and the importance of maintaining a healthy and satisfactory sexuality appear to influence sexual satisfaction. For instance, in Haavio-Mannila and Kontula’s (1997) study, not perceiving sex to be important led to dissatisfaction among women. Similarly, Thomas et al. (2015) identified a strong relationship between the importance of sex and sexual satisfaction, where higher importance correlated with higher ratings of sexual satisfaction, although they did not analyze this relationship in detail. On the other hand, Juang and Knight (2018) found that the importance of sex played a mediating role in the relationships between gender, age, partner status, and sexual distress, observing that the attitude toward sex explained the differences in sexual distress. Our study reveals an even more complex relationship where the importance assigned to sexuality mediates the relationship between behavior and sexual satisfaction.

Furthermore, Kolodziejczak et al. (2021) examined the importance that individuals place on sexuality and discovered that in late middle age, later-born cohorts assigned slightly greater value to sexuality compared with adults of the same age from earlier cohorts interviewed 20 years previously. The authors suggested that sociocultural and historical factors, such as reduced gender gaps, could contribute to these differences. Additionally, Stark et al. (2015) found that gender differences in the importance attributed to sex were smaller compared to other sexual motivations. Although these studies did not analyze SSE or sexual satisfaction, nor their interrelationships, they exemplify the growing recognition of this construct. Our results, coupled with the findings of these studies, underscore the increasing relevance of this variable, emphasizing the need to consider it when studying correlates of sexual satisfaction, especially when assessing women’s sexuality.

Despite the contributions of this study, it is important to acknowledge certain limitations that warrant attention in future research. This study focused on exploring specific aspects of sexuality in women during emerging and middle adulthood. Consequently, the findings should be interpreted exclusively in relation to this specific population, as these women may not share common life circumstances with other societal groups (e.g., women of different ages, single mothers, and women with disabilities). For these reasons, future studies should aim to replicate these findings with broader and more heterogeneous samples. The finding that the participants also share similarly high levels of sexual satisfaction also highlights the need for further research with more heterogeneous samples. Moreover, variables such as political and spiritual orientation, partner status and type (sporadic vs. committed), dyadic factors (e.g., history, experiences, and satisfaction with romantic relationships), educational level, sexual identity, knowledge and sexual attitudes, and gender norms were not considered in the analyses. Future investigations should explore the influence of these variables on the relationships of interest. The cross-sectional and correlational design of this study represents another limitation. Moreover, future studies should also explore other potential indirect effects, including effects of simple moderation, mediated moderation, moderated mediation, or serial or parallel multiple mediation with more mediating variables (Hayes, 2012), as well as other possible relationships between variables (e.g., type models proposed in PROCESS). Similarly, the role of these intervening variables in each of the satisfaction dimensions (i.e., individual and partnered, and actual and desired satisfaction) should be explored.

Despite these limitations, the present study has opened new lines of research, particularly regarding a *positive* perspective on sexuality and, more specifically, female sexuality. Historically, female sexuality has largely been overlooked, misinterpreted, or dismissed (e.g., Schmidt-Kirkpatrick, 1980; Amaro et al., 2001; Lamb, 2010; Gavey, 2012; Tolman, 2012; Bowman, 2014; Bass, 2016). The study of women’s sexuality offers valuable insights for advancing research in the field and developing interventions tailored to the circumstances, opinions, experiences, interests, and needs of this population. Such interventions should be directed toward promoting sexual health, including all dimensions of sexual satisfaction. This approach would extend beyond an approach to female sexuality and sexual wellbeing focused on reproductive issues or the absence of pathology or disease (e.g., sexual dysfunction).

## 5 Conclusion

In conclusion, this study highlights the substantial contributions of both sexual behaviors and the perceived importance of maintaining an active and satisfactory sexual life to the overall sexual satisfaction of women, particularly when considering their SSE beliefs. By shedding light on the direct and indirect mechanisms through which sexual efficacy self-perceptions impact sexual satisfaction, this research adds valuable insights to existing knowledge concerning the psychosocial correlates of sexual satisfaction in women during early and middle adulthood.

## Data availability statement

The raw data supporting the conclusions of this article will be made available by the authors, without undue reservation.

## Ethics statement

The studies involving humans were approved by the Comité de Ética en Investigación Humana (CEIH), Facultad de Medicina, Universidad de Granada. The studies were conducted in accordance with the local legislation and institutional requirements. The participants provided their written informed consent to participate in this study.

## Author contributions

AO-B: Conceptualization, Data curation, Formal analysis, Investigation, Methodology, Software, Validation, Visualization, Writing – original draft, Writing – review & editing. RL-M: Data curation, Visualization, Writing – review & editing. RG-P: Writing – review & editing. AL-G: Writing – review & editing. DG-I: Conceptualization, Data curation, Formal analysis, Investigation, Methodology, Project administration, Software, Supervision, Validation, Visualization, Writing – review & editing.
